# Epidemiology and Outcome of Self-Inflicted Burns at Pakistan Institute of Medical Sciences, Islamabad

**Published:** 2014-07

**Authors:** Muhammad Saaiq, Bushra Ashraf

**Affiliations:** 1 Department of Plastic Surgery and Burn Care Centre, Pakistan Institute of Medical Sciences (PIMS), Islamabad, Pakistan;; 2 MCH Centre, Pakistan Institute of Medical Sciences (PIMS), Islamabad, Pakistan

**Keywords:** Burn, Suicide, Burn, Injury, Pakistan

## Abstract

**BACKGROUND:**

Self-inflicted burn injuries carry considerable mortality and morbidity among otherwise fit young individuals. This study assessed the epidemiologic pattern and outcome of these injuries in a burn care facility in Pakistan.

**METHODS:**

The study was carried out at Pakistan Institute of Medical Sciences (PIMS) Burn Care Centre in Islamabad over a period of 2 years. It included all adult patients of either gender, aged over 14 years who presented as cases of burn suicides and attempted burn suicides during the study period. Convenience sampling technique was employed. The sociodemographic profile of the patients, motives underlying the act of self-immolation, any underlying psychiatric illness, alcohol abuse, total body surface area (TBSA) burnt, depth of burn injury, associated inhalation injury, duration of hospital stay, and mortality were all recorded.

**RESULTS:**

Seventy five patients (80.64%) were female while 18 patients (19.35%) were male. The overall mean age was 26.89±6.1 years (range=15-52 years). The affected TBSA ranged from 15%-100% with an overall mean of 69.30±25.42%. The hospital stay ranged from 1-37 days with a mean of 7.16±6.60 days. Marital conflicts constituted the most frequent motive underlying the suicidal attempts (n=57; 61.29%) followed by failed love affairs (n=9; 9.67%). There was an overall mortality of 84.95%. The most common sufferers of self inflicted burn injuries were young, married, illiterate housewives who were resident of rural area. Getting marriage was the most common triggering cause for such injuries.

**CONCLUSION:**

There is need to institute appropriate preventive measures to address the issue in a national perspective.

## INTRODUCTION

Burn injury as a major cause of death and disability has a high cost in health care with an increasing trend in mortality and morbidity in patients.^1^^,^^2^ Healing in burn wounds is still a challenge even some medications were introduced in the literature. So every effort is done to provide a shorter in-patient care for burn patients.^3^ *Pseudomonas aeruginosa* as an important cause of nosocomial infection may result into septicemia and death in burn patients denoting to its public health importance.^4^ So it seems that there is a need for burn dressings and more effective therapeutic medications for burn patients. Several products were shown to have therapeutic efficacy and to be with less toxicity when compared with synthetic drugs.^5^^-^^8^

Burn suicide (self-immolation or self-inflicted burns) constitutes one of the most bizarre suicidal approaches that continue to plague humanity even today. Self-immolation represents one extreme of the burn injury spectrum while the other extreme is constituted by assault burn injuries.^9^^-^^12^

Globally, the reported rates of burn suicides vary considerably from country to country and centre to centre. These account for less than 1% of all suicides in high-income countries (such as the United States), however these figures are alarmingly higher in many low and middle income countries like Iran, India, Sri Lanka, Iraq and Afghanistan. Burn suicide accounts for up to 40% of all suicidal deaths in some parts of the developing world and as high as 71% of the suicides in their specific locales.^10^^-^^20^

Generally, the burden of burn injuries is disproportionately shared across the globe with over 95% of burn deaths occurring among the developing nations.^21^ This imbalance is further compounded by the menace of burn suicide which predominantly affects the developing nations. Overall, burn suicides account for 2-36.6% of all burn injury admissions worldwide.^21^^-^^24^ The present study was carried out to document the prevalent sociodemographic pattern and outcome of self-inflicted burns in our centre at Islamabad, and hence generate evidence base to better address this menace in a national perspective.

## MATERIALS AND METHODS

This observational study of self-inflicted burns spanned over a period of two years from June 2010 to May 2012. It was carried out at Burn Care Centre of Pakistan Institute of Medical Sciences (PIMS) in Islamabad, Pakistan and included all adult patients of either gender, aged over 14 years who presented as cases of burn suicides and attempted burn suicides during the study period. 

Convenience sampling technique was employed. Those cases were excluded from the study where either the patient or the accompanying attendants refused to be consent for inclusion in the study. Self-inflicted burn was defined as the purposeful act of self burning with suicidal intention. As the study was an observational one and did not involve any new intervention, it was conducted in accordance with the Declaration of Helsinki of 1975, as revised in 2008 and anonymity of the participants was guaranteed. Informed consent was taken from all the patients or their attendants for inclusion in the study. 

Initial assessment and diagnosis of burn injury was made by a thorough history, physical examination and ancillary investigations. The total body surface area (TBSA) of burn was calculated by employing ‘rule of nines’. All the patients were admitted for indoor management. Data collection was performed with the help of a comprehensively designed proforma that encompassed all relevant epidemiologic and clinical variables of interest. The sociodemographic profile of the patients (i.e. age, gender, marital status, educational status, employment status, rural versus urban origin), motives underlying the act of self-burning, any underlying psychiatric illness, alcohol abuse, TBSA burnt, depth of burn injury, associated inhalation injury, duration of hospital stay, and mortality etc. were all recorded on the proforma. 

Serial personal interviews with the patient and the accompanying attendants (i.e. spouse, parents, in-laws, siblings, family members and other accompanying supporting neighbors) in separate sessions (through the entire course of management) were carried out to ascertain the circumstances and dynamics surrounding the act of self-burning. The main outcome measures were mortality and length of hospital stay. 

As per protocol of our centre, all these patients were managed according to standard management protocols of burn injury. Additionally, the medico-legal issues surrounding the act of burn suicide and attempted burn suicide were dealt with by the medico legal officer of the hospital. 

The data were subjected to statistical analysis using statistical package for social sciences (SPSS) (Version 17, Chicago, IL, USA) and various descriptive statistics were employed to calculate frequencies, percentages, means and standard deviation. The numerical data such as age, TBSA burnt and duration of hospitalization were expressed as mean ± standard deviation while the categorical data such as the motives for suicides and various associated risk factors were expressed as frequency and percentages. The percentages were compared by employing Chi-Square test and a P value of less than 0.05 was regarded statistically significant. 

## RESULTS

During the study period, a total of 93 patients fulfilled the inclusion criteria. Out of these, 75 (80.64%) were females while 18 (19.35%) were males. The age of the patients ranged from 15 to 52 years with an overall mean age of 26.89±6.1 years. The mean age for females was slightly less than that for the males (i.e. 26.26±6.35years versus 29.50±4.4years).

The affected TBSA ranged from 15 to 100% with an overall mean of 69.30±25.42%. The involved TBSA was significantly greater among those who died when compared with those who survived the suicidal attempts (i.e. 76.83±19.25% versus 26.78±7.75%; P<0.05).

The hospital stay ranged from 1-37 days with a mean of 7.16±6.60 days. The hospital stay was significantly shorter among those who died than those who survived. (i.e. 5.31±4.53 days; range= 1-21 days versus 17.57±6.94; range=10-37 days) (P<0.05).

The various sociodemographic and injury characteristics found among the patients were summarized in Table 1. Getting marriage was the most frequent motive underlying the suicidal attempts (n=57; 61.29%) followed by failed love affairs (n=9; 9.67%) as shown in Table 2. 

**Table 1:** The various sociodemographic and injury characteristics observed among the patients (n=93).

**Variables**	**Number (%)**	**P value (%)**
GENDER		
Females	75 (80.64)	<0.001*
Males	18 (19.35)	
AGE		
Upto 35 years	90 (96.77)	<0.001*
>35 years	3 (3.22)	
TBSA burnt		
>50%	67 (72.04)	<0.001*
<50%	26 (27.95)	
Depth of Burn Injury		
Deep	86 (92.47)	<0.000*
Superficial/Mixed	7 (7.52)	
Inhalation Injury		
Present	59 (63.44)	<0.05*
Absent	34 (36.55)	
Marital Status		
Married	66 (70.96)	<0.001*
Un-married	23 (24.73)	
Widowed/ Divorced	4 (4.30)	
Educational Status		
Illiterate	79 (84.94)	<0.001*
Literate/educated	14 (15.05)	
Employement Status		
Housewife	58 (62.36)	<0.001*
Others	35 (37.63)	
Place of Living		
Rural areas	86 (92.47)	<0.001*
Urban areas	7 (7.52)	

* Significant P <0.05

**Table 2:** Motives triggering burn suicides among the patients (n=93).

**Motives**	**Number of patients (%)**
Marital conflicts	57 (61.29)
Failed love affairs	9 (9.67)
Political protests	2 (2.15)
Known depression	1 (1.07)
Alcohol use	1 (1.07)
Unknown/ Undisclosed reasons	23 (24.73)

**Fig. 1:**
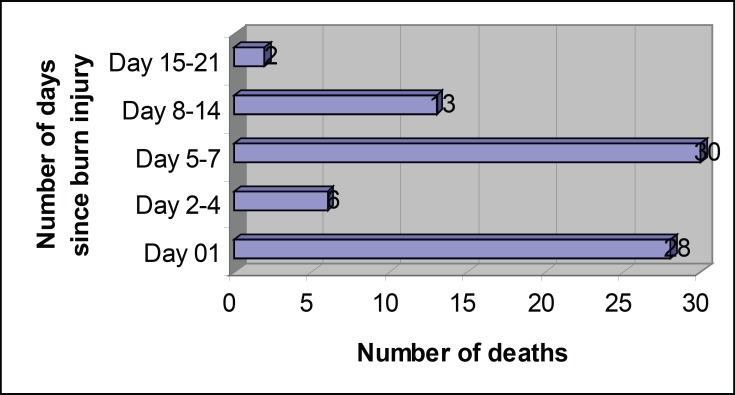
Number of deaths in relation to the number of days following burn injury (n=79).

There were 79 deaths accounting for an overall mortality rate of 84.95%. First peak of high number of deaths (n=28; 35.44%) was observed during the first 24 hours, followed by the second high peak in number during 5-7 days after sustaining burn injury (n=30; 37.97%). The time intervals between sustaining burn injury and deaths are shown in Figure 1.

## DISCUSSION

Self-inflicted burns are prevalent in Pakistan, however there is scarcity of published literature on the issue. Most of the recently published local studies are either focused on the epidemiology of burns in general or pertain to some particular aspect of burn injury management.^24^^-^^28^

Hence our study serves to represent the sociodemographic pattern and outcome of self-inflicted burn injuries in our country. Owing to lack of burn suicides registry in our country, the exact incidence of such injuries is difficult to estimate, however our study with 93 cases over two years period signifies the fact that it is one of our prevalent but neglected medical issues. In our series, the female patients were in overwhelming majority. 

Predominant involvement of females in burn suicides is also reported by most of the published studies from developing countries including Iran, India, Sri Lanka, Iraq, Uzbekistan, and Afghanistan.^13^^,^^16^^-^^20^^,^^29^^-^^35^ In sharp contrast to the female predominance in burn suicides in the developing world, studies from the European countries, Australia and North America have reported more frequent involvement of males than females.^36^^,^^37^

In our series, adult females in their second and third decades of life constituted bulk of the patients. This conforms to most of the published studies from countries like Iran, India, and Iraq,^11^^-^^18^^,^^38^^-^^40^ however Chan et al.^41^ from Hong Kong, Seoighe et al.^42^ from Ireland and several other studies from the West and Australia have reported more frequent involvement of higher age groups.^43^^-^^46^ In our series, the bulk of patients was constituted by married women. This observation is in conformity to what is reported by most of the published studies from other developing countries.^11^^-^^19^^,^^23^^,^^29^^-^^33^^,^^47^^-^^53^

Marital conflict and failure in love affairs were the most frequently admitted underlying motives for self inflicted burns in our patients. This observation conforms to many reported studies from Iran, India, Afghanistan and Iraq. There exists a considerable body of published literature on self-inflicted burns from Iran wherein marital and interpersonal conflicts, and various forms of oppression and violence against women have been recognized as the triggering factors. Such domestic and spousal disharmony is further compounded by low family income, unemployment of the spouses, and low levels of literacy among women.^11^^-^^13^^,^^16^^,^^29^^-^^31^^,^^40^^,^^47^^,^^53^^-^^55^

As the victims are physically, emotionally, or mentally tortured by their husband, husband’s family, or even their own family members, the young women’ act has been referred to as a ‘‘cry for help’’ with the victims finding this self-humiliating act as a means of both escaping from intolerable conditions as well as speaking out against their abuse.^11^^,^^16^^,^^29^ Groohi et al.^30^ identified quarrel with a family member/relative and marital conflicts as the major factors precipitating suicidal burns. 

Maghsoudi et al.^39^ reported domestic violence against women as one of the precipitating factors for suicidal burns. In contrast to the societal picture in Pakistan and Iran, the social dynamics in India are yet different and young wives commit burn suicides in response to the pressure of dowry disputes. Additionally, the young bride is typically living in a joint family where she is expected to perform majority of the household chores. She is not given say over choice of husbands. Historically in the past, the Indian widow women were forced to set themselves ablaze at the funeral of their husbands in the name of “*sati”.*^17^^,^^56^

In Afghanistan, the situation is even more sinister with majority of burn suicides occurring among women as a reaction to their gross social deprivation. For instance, women are strictly under the authority of the father or husband and cannot have the opportunity to assert economic and social independence. They are also faced with inhuman acts of Badal, wherein women are exchanged for material goods and money.^20^^,^^57^^,^^58^

Al-Zacko et al. in Iraq have related the burn suicides among women to the major changes happening at their age; the transition from youth to adulthood and adolescent marriages, with all the responsibilities coming particularly on young girls who are quite often ill-prepared to play the role of housewives.^19^

Contrary to all the aforementioned observations in studies from the developing economies, the situation in the developed and Western nations is different and burn suicides have been reported to be more common among men, slightly of higher age group, and among individuals with co-morbid substance abuse, and psychiatric and adjustment disorders.^37^^,^^41^^,^^46^^,^^59^^-^^63^

In our series, majority of the patients were illiterate. This observation conforms to the published literature from developing countries like Iran and India.^11^^-^^17^^,^^29^^-^^32^^,^^39^^,^^46^^,^^53^^-^^58^ One possible explanation for this is that education greatly improves the personality of the individual and enhances her understanding of daily life situations and family dynamics. Also education empowers women who can better voice their concerns about any injustices and get their rights more efficiently. This helps to avert the possibility of self-defeating behavior and indulgence in suicidal activities.^46^^,^^53^^-^^58^

In our series, majority of the patients were from rural areas. The published literature has also reported a higher rate of suicidal burns among the rural population than the urban population.^11^^-^^16^ In our series, the bulk of patients was constituted by those with over 50% TBSA burnt. Our observation conforms to most studies on self-immolation which have reported a greater total body surface area affected, with higher incidence of smoke inhalation injury, and with more difficult course of illness.^17^^-^^19^^,^^64^^,^^65^

The mortality rate in our series was 84.95%. Other published studies have also reported high mortality rates, ranging from 25 to 90%.^17^^-^^19^^,^^64^^,^^65^ In developing countries, the course and outcome is reported to be generally more severe with most women not reaching the hospital and most dying within the first 24 h.^17^^,^^66^ Two of our patients presented with self inflicted burns in the court premises as a symbolic measure of protest. Political protest is a relatively rare motivation for self-immolation. Published literature has similar examples of political protesting with self-immolation.^34^ Such protests often receive vast coverage by the mass media and trigger political and civil unrests. 

Not surprising we faced great difficulty in extracting true story of the events underlying burn suicides among our patients. At the very outset, the patients as well as the attending family members denied the case as one of self-inflicted burns and insisted on its being an accidental event. They often narrated different false stories, which an experienced burn care provider would prudently tamper with clinical judgments of the circumstantial evidences surrounding the suicidal event plus the typical stereotyped distribution of the burn injuries. Several published studies have expressed similar observations of their patients’ behavior and related them to social and religious factors and the tendency to hide the precipitating social stressors.^9^^,^^67^

Our study has some strength as well as presents some limitations. It is the first local study which has tried to identify factors associated with self inflicted burn injuries among adults in Pakistan and hence established evidence base regarding this important health issue. As it was a hospital based study, we could not measure the exact incidence of burn suicides and attempted burn suicides at our typical population level. Although all the surviving individuals were routinely referred to psychiatrist, our study did not include their long term follow up to document the residual post-burn physical and psychiatric morbidity amongst them. We recommend the conduct of further local studies to improve upon these limitations.

The most common sufferers of self inflicted burn injuries were young, married, illiterate, housewives belonging to a rural background. Marital conflicts constituted the most common triggering cause for such injuries. These injuries carry considerable mortality and morbidity of prolonged hospitalization among otherwise fit young individuals. Our study highlights the gravity of this major but neglected catastrophe in our resource constrained economy. There is dire need to institute appropriate measures to address the issue in a national perspective.

## CONFLICT OF INTEREST

The authors declare no conflict of interest. 

## Footenote

Please cite this paper as:

Saaiq M, Ashraf B. Epidemiology and Outcome of Self-Inflicted Burns at Pakistan Institute of Medical Sciences, Islamabad. World J Plast Surg 2014;3(2):107-114.
